# Asian Hornet, *Vespa velutina* Lepeletier 1836 (Hym.: Vespidae), Venom Obtention Based on an Electric Stimulation Protocol

**DOI:** 10.3390/molecules27010138

**Published:** 2021-12-27

**Authors:** Xesús Feás, Carmen Vidal, M. Pilar Vázquez-Tato, Julio A. Seijas

**Affiliations:** 1 Academy of Veterinary Sciences of Galicia, Edificio EGAP, Rúa Madrid, No. 2-4, 15707 Santiago de Compostela, (A Coruña), Spain; 2 Fundación Instituto de Investigación Sanitaria de Santiago de Compostela (IDIS), Hospital Clínico, Edificio D, 1ª Planta, Travesía da Choupana S/N, 15706 Santiago de Compostela, (A Coruña), Spain; 3 Servizo de Alergoloxía, Área Sanitaria de Santiago de Compostela e Barbanza, Hospital de Conxo, Rúa de Ramón Baltar S/N, 15706 Santiago de Compostela, (A Coruña), Spain; carmen.vidal.pan@sergas.es; 4 Área de Medicina, Departamento de Psiquiatría, Radioloxía, Saúde Pública, Enfermaría e Medicina, Facultade de Medicina e Odontoloxía, Rúa San Francisco S/N, 15782 Santiago de Compostela, (A Coruña), Spain; 5 Departamento de Química Orgánica, Facultad de Ciencias, Universidad de Santiago de Compostela, Alfonso X el Sabio, 27002 Lugo, Spain; pilar.vazquez.tato@usc.es

**Keywords:** Asian hornet, *Vespa velutina*, venom, electrical, stimulation, allergy, stings, invasive species

## Abstract

The yellow-legged Asian hornet (*Vespa velutina* Lepeletier 1836 (Hymenoptera: Vespidae)) is naturally distributed in China, Southeast Asia, and India; however, recently it has been detected outside of its native area, confirmed as being established in South Korea, Europe, and Japan. Health risks and deaths caused by the invasive *Vespa velutina* stings have become a public health concern, being the most common cause of anaphylaxis due to hymenopterans in some European regions. This in turn has led to increased demand from medical practitioners and researchers for *Vespa velutina* venom for diagnostic and therapeutic purposes. In this study, a straightforward, quick, and inexpensive method for obtaining *Vespa velutina* venom by electric stimulation is described. The venom extracts were analyzed by nuclear magnetic resonance spectroscopy (^1^H-NMR). The availability of *Vespa velutina* venom will lead to improved diagnostic and therapeutic methods, mainly by venom immunotherapy (VIT), in patients allergic to this invasive species.

## 1. Introduction

Invasive alien species (IAS) are plants, animals, pathogens, and other organisms that are introduced and/or spread outside of their natural geographic range and which may cause severe ecological, economic, and social impacts on the invaded environments. The European Union experiences annual damages worth €12 billion as a result of IAS effects on human health, damaged infrastructure, and agricultural losses [1]. Recently it was estimated that IAS have cost North America $2 billion per year in the early 1960s to over $26 billion per year since 2010 [2] and that the economic cost of IAS has been $1.288 trillion over the past 50 years [3]. Over 100 examples of IAS that affect human health, sometimes with devastating effects on our livelihood, have been described and documented around the world [4].

Several IAS insect species have migrated in the last decade to Galicia, located on the north-western end of the Iberian Peninsula, and successfully colonized and spread, resulting in a broad range of consequences to recipient ecosystems and, thereby, human society [5]. Of these IAS, the yellow-legged Asian hornet (*Vespa velutina* Lepeletier 1836 (Hymenoptera: Vespidae)) was detected in Galicia in 2012.

*Vespa velutina* is naturally distributed in Southeast Asia, India, and China. It was first detected outside of its native habitat in South Korea in 2003 [6], in southwestern France in 2004 [7], on islands of Japan in 2012 [8], and on the Japanese western mainland in 2015 [9].

It was soon recognized as a pan-European threat after being detected in the province of Navarra, northern Spain (2010); in the north-western province of Minho in Portugal (2011); Belgium (2011); Italy (2012); Germany (2014); the Netherlands (2018); Majorca in the Balearic Islands (2015); and England and the Channel Islands (2016) [5].

The species has become a major concern to apiculture and industries relying on pollination, given that the diet of these hornet colonies is predominantly based on honey bees and other insects. In Galicia, the annual cost of lost production is estimated at more than 4.5 million euros. The *Vespa velutina* could be responsible for the loss of 65% of the bee colonies in infested areas [10].

*Vespa velutina* is not only a problem for beekeepers and their industrious flying insects, since other agricultural sectors such as fruit producers and viticulturists are also impacted. Despite this, the medical-veterinary potential of *Vespa velutina* should also be outlined. In the Aculeates, the defining feature is that the egg-laying ovipositor is modified to form a sting. Hymenopteran insects are not predisposed to assault and sting humans, however, social hornets, wasps, and bees have a large defensive response to any risk to the colony. The evolution of the venom system shows to have further developed to cause pain and increase the immune response in humans and different vertebrate predators [11]. Due to its habits, abundance, and wider distribution, the risk that the IAS *Vespa velutina* represents for human health is incomparable with other native species of hymenoptera [12,13].

Currently, in invaded areas such as Spain and South Korea, there is an increase in the number and severity of reactions in patients exposed to invasive species *Vespa velutina* venom. Clinical data confirm anaphylaxis to *Vespa velutina* to be a significant emerging problem. Anaphylaxis to *Vespa velutina* has increased exponentially from the first case in 2015 in our area, where it represents the most common form of Hymenoptera anaphylaxis today. More than three-quarters of incoming patients thatreported Hymenoptera anaphylaxis between December 2017 and June 2019 identified *Vespa velutina* as the insect responsible for the reaction. The number of incident cases of anaphylaxis caused by other Hymenoptera (*Vespula* species and *Apis mellifera*) remained stable in those years [14]. In the University Hospital in Santiago de Compostela, Spain, covering an area of 500,000 people, a total of 292 patients were receiving venom allergen immunotherapy in 2020. In South Korea, from 2010 to 2014, there were 483,233 calls requesting the removal of wasp nests and the stings of Hymenopterans caused 78,860 injuries and 49 deaths, with *Polistes rothneyi koreanus* Vecht and *Vespa velutina* being the most prevalent sources. The total medical costs associated with the stings of hornets and wasps over a 5-year period were approximately 3.2 million dollars [15]. In Galicia, the number of calls received by the emergency services “112 Galicia” related to “incidents” with the *Vespa velutina* totalled 42,901 in a period of 3 years (2015–2017) [5]. Two colonies of the *Vespa velutina* in Galicia were detected in 2012, 17 in 2013, 769 in 2014, 5022 in 2015, and 10,642 in 2016; in 2019, around 25,000 colonies were destroyed, settling widely in urban spaces [12].

The medical community is requesting *Vespa velutina* venom extracts to aid diagnosis and treat allergy and/or anaphylaxis through immunotherapy [16,17]. Venom immunotherapy is the standard of care for people with severe reactions and has been shown to reduce the risk of future anaphylactic events and risk of death [18]. The availability of locally appropriate venom extracts must be ensured in order to diagnose and treat hymenopteran venom allergies through immunotherapy [19]. The key motivation behind the present research was to obtain extracts of venom from the invasive species *Vespa velutina.* It therefore has a very important practical application at the clinical level and could ultimately improve the lives of those highly reactive to the sting of the invasive species *Vespa velutina* and cannot “yet” receive immunotherapy due the lack of readily available and reliable sources of venom extracts.

## 2. Results and Discussion

Detailed information with all aspects required for finding, collecting, and properly handling *Vespa velutina* specimens, as well as apparatus and methods used for venom extraction, are described in the present work.

### 2.1. Collection and Identification of the Insects

**Figure 1:**
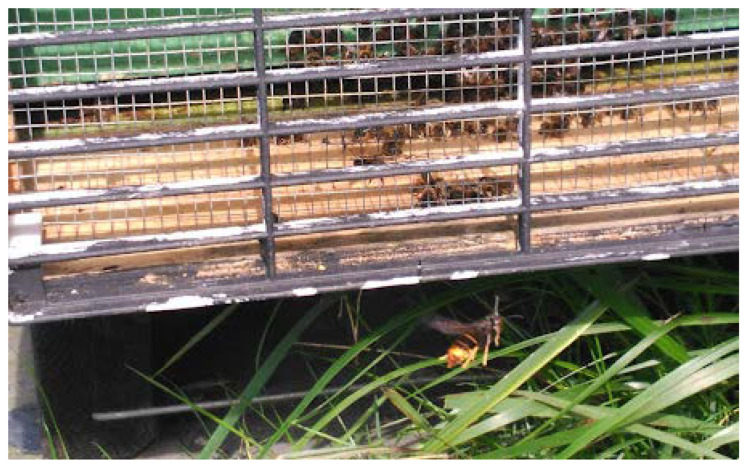
A female *Vespa velutina* specimen in static flight in front of the beehive’s entry at the apiary, located in San Pedro de Viveiro at the province of Lugo (Galicia, Spain).

At first glance, the sampling of the *Vespa velutina* specimens seems trivial. *Vespa velutina* is almost ubiquitous in the invaded areas. However, sampling insects requires knowledge of their biology, preferred habitats, and activity patterns. Apiaries are hot spots for the collection of *Vespa velutina,* as they are concentrated, abundant, and easy to capture [20]. *Vespa velutina* are notorious honey bee hawkers. They fly continuously and hover around the beehive entrance at a distance of 10–40 cm (Figure 1), and hunt in flight by intercepting arriving or departing honey bees to the beehive, grabbing the foragers with their outstretched legs.

The target insect is not easily confused with any other hymenopteran species of hornet, bees, and wasps normally present at the apiaries in different areas of Europe, such as the European hornet, *Vespa crabro* Linnaeus, 1758; *Vespa orientalis* Linnaeus, 1771; *Bombus* spp; *Vespula* spp; and/or *Polistes* spp. The *Vespa velutina* can be clearly differentiated because of its unique dark colour pattern, which is mostly black. Moreover, the *Vespa velutina* has the fourth abdominal segment almost entirely orange–yellow; is smaller than the native European hornet, *Vespa crabro* Linnaeus, 1758; and possesses yellow-tipped legs (Figure 1).

The availability of arthropod venom still remains as the main barrier in arthropod toxinology. The difficulties in obtaining sufficient amounts of arthropod venom are either due to scarcity of the given venomous animal and/or the difficulties in the collection of its venom [21]. The distinctive *Vespa velutina* honey bee-capture behavior allows the pinpointing of apiaries as an ideal place, to catch enough live specimens for posterior venom extraction. The *Vespa velutina* colonies are typically at their maximal size in late summer and/or early autumn, with thousands of individuals in their nests, increasing their presence at the apiaries. *Vespa velutina* specimens were collected in an apiary in Viveiro, around 20 km away from the one of the first two original entry points of *Vespa velutina* detected in Galicia in October 2012 (GPS, UTM; X: 632451, Y: 4834800) [5].

**Figure 2:**
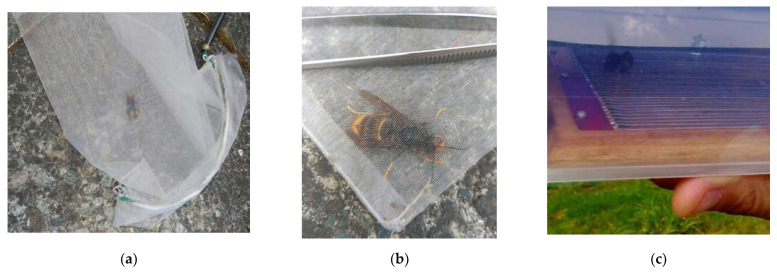
A captured female *Vespa velutina* specimen by aril netting: (**a**) Walking up into the net bag; (**b**) Trapped in a fold of the tip net bag; (**c**) Inside the designed chamber for venom extraction.

Based on our field experiences and long term observations and research of the *Vespa velutina,* we carried out an effective sampling and easy transferral of the *Vespa velutina* from the apiary with a net to the venom extraction box (Figure 2a–c). Since *Vespa velutina* specimens are abundant in static flight in front of the beehives at the apiary, a fast horizontal swing of the net allows for the effective capture of this insect. Efficient use of a net to capture the *Vespa velutina* is gained only with experience. With a little practice, this becomes quite simple to perform. Because the hornets display positive phototropism, they facilitated their transfer from the net to the venom extraction box by moving through the black tube towards the light.

### 2.2. Electric Stimulation

The *Vespa velutina* venom extraction chamber is lightweight (1.3 kg) and mobile, so it can be used out in the field. The electrical venom collection device is an all-in-one solid, smart compact, working with automatic tuning where a microprocessor monitors and adjusts pulses, based on humidity, number of insects, how long the device has been running, the battery level, and the venom collector’s overall condition. The access to the on/off button allows users to operate the device safely. Every 50 s, the device pauses for 10 s. After 40 min of work time, the device turns off automatically. That was the maximum time for *Vespa velutina* venom collection in one session, for a total of 10 individuals.

**Figure 3:**
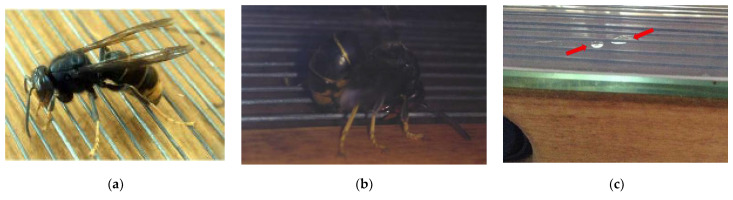
A female *Vespa velutina* specimen in the milking grid: (**a**) Resting, venom collection device off; (**b**) Bending the tip of their abdomen downward and stinging after receiving an electrical shock, venom collection device on; (**c**) Deposited liquid globules of venom over the glass plate (red arrows).

**Figure 4:**
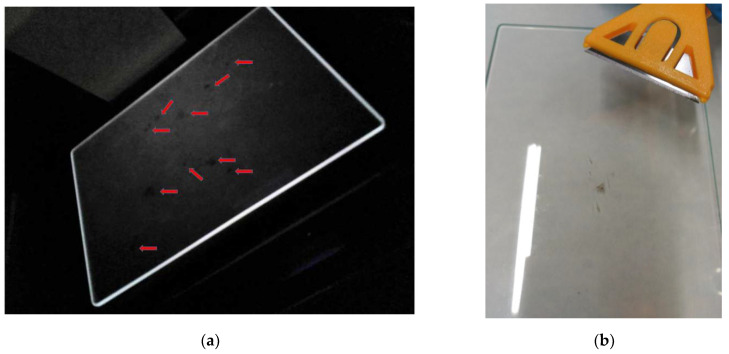
At the laboratory, the removed glass plate from the venom collector device after *Vespa velutina* electric stimulation (**a**) under UV light, showing venom-dried spots (red arrows); (**b**) scrape of the venom.

A close visual inspection of the electrical venom collection device (Figure 3a,b) allows us to observe liquid globules on the over the glass plate (Figure 3c), where globules crystallize or dehydrate quickly with exposure to the air. Once transported to the lab, in less than 2 hours, no liquid is observed on the plate, although an inspection under ultraviolet light does reveal small spots on the glass (Figure 4a). The dried venom was removed from the glass plates carefully with a razor (Figure 4b).

At a lab-scale level, there are well tested and widely accepted techniques for obtaining venom from several hymenopterans, such as: (i) *Polybia paulista* Ihering, 1896 [22]; *Apis mellifera carnica* Linnaeus, 1758 [23]; and *Vespa affinis* Linnaeus, 1764 [24]; and (ii) *Vespula maculifrons* Buysson, 1905; *Vespula germanica* Fabricius, 1793; *Vespula vulgaris* Linnaeus, 1758 [25]; *Polistes annularis* Linnaeus, 1763; *Polistes carolina* Linnaeus, 1767; *Polistes exclamans* Viereck, 1906; *Polistes fuscatus* Fabricius, 1793; *Polistes instabilis* Saussure, 1853; and *Vespula germanica* Fabricius, 1793 [26]; *Dolichovespula maculata* Linnaeus, 1763; *Polistes annularis* Linnaeus, 1763; and *Vespula vulgaris* Linnaeus, 1758 [27].

They are based on dead, frozen insects, where the whole sting apparatus needs to be dissected, using microsurgical forceps, and the obtained venom sacs are basically: (i) manually extracted from the separated venom sacs, i.e., by gentle squeezing, or (ii) homogenized and/or just pooled to collect the liquid fraction by centrifugation.

The above existing venom extraction protocols [22,23,24,25,26,27] are followed to obtain actual *Vespa velutina* venom [28,29,30,31]. The venom reservoir of the *Vespa velutina* is about 1 mm in length, white and transparent, [28,29] and the microdissection of the venom reservoir, usually by the precise manipulation of specialized needles, requires a high degree of operator skills. This makes the manual extraction of venom a tedious, laborious, and time-consuming task.

Li et al. demonstrated that honey bee venom extracted manually is different from venom extracted using electrical stimulation, and these differences may be important in their use as pharmacological agents [32]. The venom extracted manually is contaminated by non-toxin proteins that may leak from the gland tissue that is cut/disrupted during venom collection. Data reported by Li et al. showed that the toxin component in the venom of manual collection has significantly lower content than venom from electrical stimulation. The three newly identified phosphorylated honey bee venom proteins in the venom extracted through the use of electrical stimulation may elicit a different immune response through the specific recognition of antigenic determinants [32]. A comparison of venom from three vespid species (*Vespula maculifrons*, *Vespula maculata* and *Vespula arenaria*) collected by venom sac extraction and electrostimulation showed potent allergens, the last one having the potential advantage of being free of contaminating tissue protein [33]. There is a scope for future research, using modern technology, to assess the potential differences between a vespid venom, in this case, venom from *Vespa velutina*, obtained by both methods: manual extraction and by electrical stimulation. Venom from gland extracts may possess components derived from the gland’s epithelium, muscular layer, nerves, etc. Furthermore, it was suggested that foreign enzymes included in the gland extracts may affect the active components of the venom [21].

**Figure 5:**
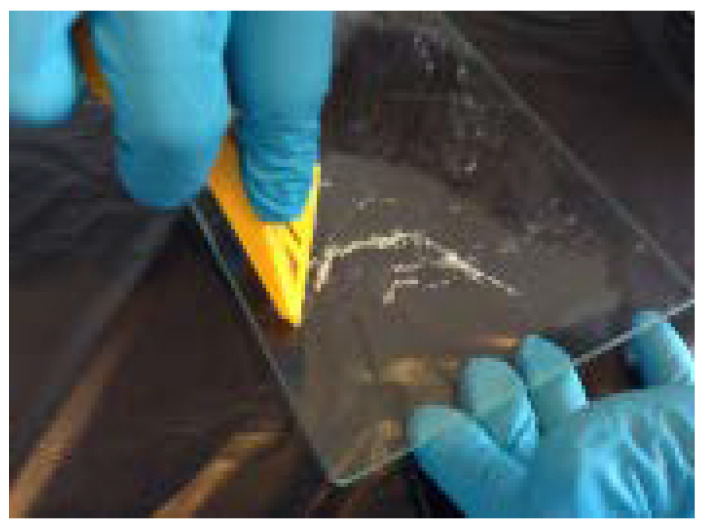
Scrape of the *Vespa velutina* venom obtained from a colony of 960 individuals from a captive nest last November 2020.

A variety of stimulation techniques have evolved and are available for the collection of venom from individual Hymenoptera as well as simultaneously from a large numbers of insects [34,35,36,37,38]. Since the first venom extraction carried out in 2017, we continued to develop the technique, adapting the venom extraction box, on account of a specific behaviour of a whole colony in their nest, and were able to obtain venom extracts by electro stimulation of several hundreds of individual hornets, obtained directly from a captive nest (Figure 5). The above allows the collection of more venom over a short time (Suplementary video 1). However, to use a live *Vespa velutina* nest for milking the venom of their inhabitants is a high risk activity, needing skill that requires specialist training, specific working places, and more resources and infrastructure.

Targeted netting to capture the insect can provide enough samples of *Vespa velutina* specimens for venom collection by electrical stimulation. However, in the specific case of social wasps, there is a long tradition of harvesting wild nests to eat larvae and pupae, as well as the use of nests in medicine recipes. Moreover, collectors have also developed practices that can be understood to some extent as incipient vespiculture [39]. Although rigorous testing is required as current Vespa hornet rearing efforts are undeveloped, research indicates the prospect of a functional year-round Vespa hornet rearing process being developed. The current biological, ecological, medicinal, and culinary motivations justify the development of true hornet vespiculture [40]. We expect that the method developed here for venom extraction of the *Vespa velutina* will prove satisfactory for collecting venom from other species of venomous arthropods and/or stinging insects, as well as reared colonies of bumblebees, wasps, and hornets. Breeding of such target insects would reduce the necessity of wild harvesting.

A prerequisite to studying the nature of the venoms is the development of methods for their collection. The collection of venom should no longer be a limiting factor as this work details a successful method to allow for the easy collection of venom. Integrating transcriptomic and proteomic analyses should provide a better understanding of: the (i) venom composition of venomous hymenoptera in particular; and (ii) mammalian immune system responses to those stinging insects. It is hoped that this will assist in any future applications of venoms into diverse biomedicine and the possible discovery and development of new pharmacological agents and other related research areas [31,41,42,43,44,45].

### 2.3. ^1^H-NMR Vespa Velutina Venom Analysis

**Figure 6:**
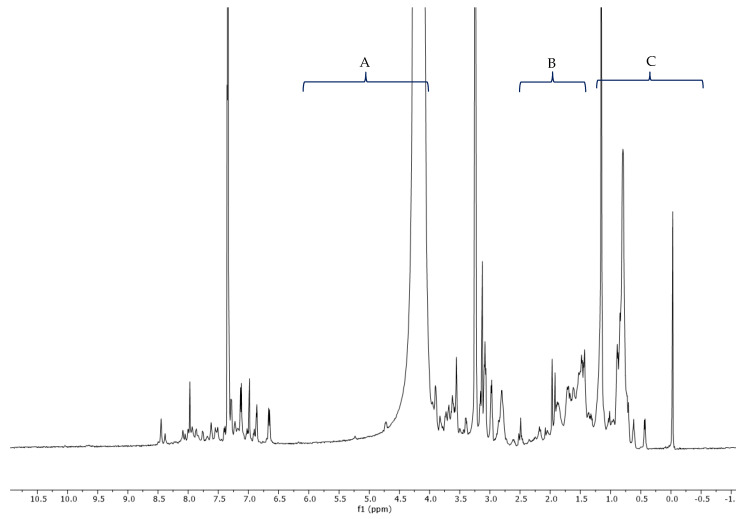
^1^H-NMR spectra (500 MHz, CD_3_OD) of *Vespa velutina* venom obtained by electrical stimulation.

**Figure 7:**
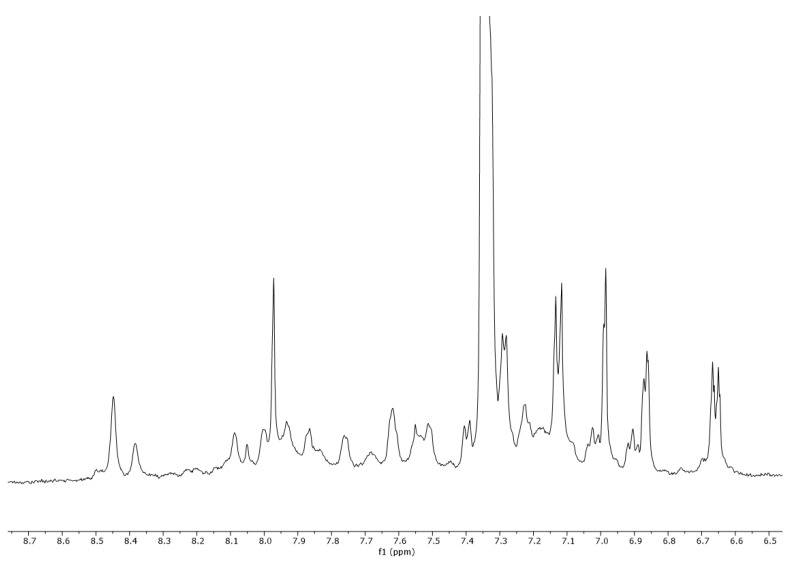
Expanded region A (δ = 6.5–8.7 ppm) of a ^1^H-NMR spectra (500 MHz, CD_3_OD) of *Vespa velutina* venom obtained by electrical stimulation.

**Figure 8:**
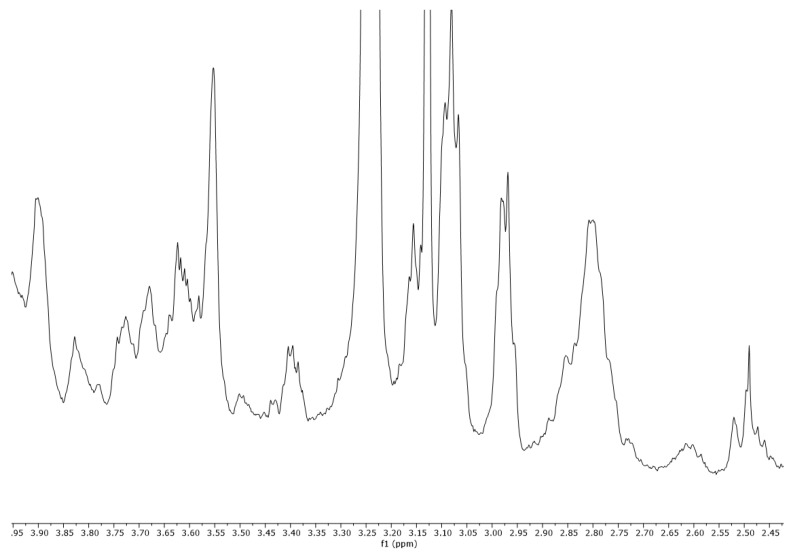
Expanded region B (δ = 2.45–3.95 ppm) of a ^1^H-NMR spectra (500 MHz, CD_3_OD) of *Vespa velutina* venom obtained by electrical stimulation.

**Figure 9:**
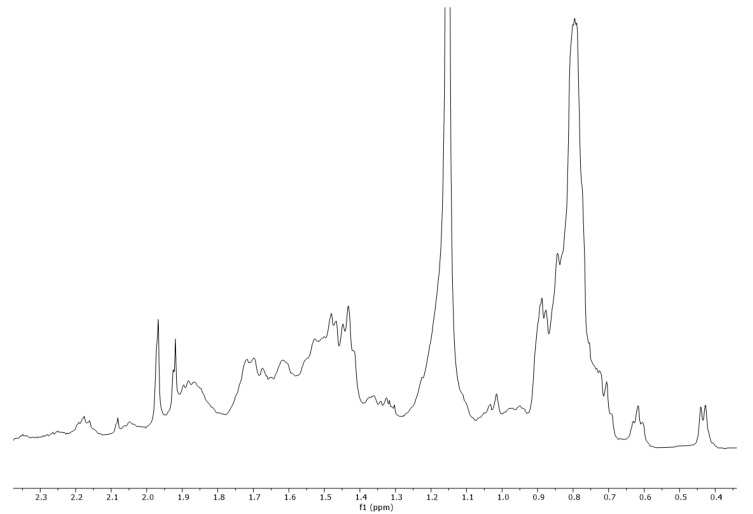
Expanded region C (δ = 0.4–2.3 ppm) of a ^1^H-NMR spectra (500 MHz, CD_3_OD) of *Vespa velutina* venom obtained by electrical stimulation.

The collected venom was dissolved in methanol-d_4_ (CD_3_OD) and analyzed by ^1^H-NMR (500 MHz). In the proton spectrum (Figure 6), three regions (A, B, and C) can be observed in the enlargements (Figure 7, Figure 8 and Figure 9). Expanded region A (δ = 6.5–8.7 ppm) shows the signals of aromatic hydrogens present in tryptophan, phenylalanine, tyrosine, and histidine.

## 3. Materials and Methods

The field study was conducted during September 2017 in one apiary (43°37′15.2″ N 7°35′26.7″ W) located in San Pedro de Viveiro (municipality of Viveiro), in the Western Mariña at the province of Lugo (Galicia, Spain). At an altitude of 177 m above sea level, the climate in the area is characterized by mildness and rainfall, as corresponds to the oceanic climate. The average annual temperature exceeds 14 °C, while the thermal oscillation is weak (10 °C), as a result of a mild winter and moderate temperatures in summer.

Adult female *Vespa velutina* specimens were obtained while using an active collecting net method at an apiary. The apiary had a total of (i) six beehives with a frontal protective module, a grid which prevents the entry of the *Vespa velutina* into the beehive; and (ii) 12 traps situated at the beehives consisted of a 15 L plastic box (width = 36.5 cm; depth = 28.5 cm; height = 18.5 cm) with four holes on the sides of each box. The bait used consisted of blueberry juice, brown beer, and wax obtained from honey bee combs.

### 3.1. Materials

#### 3.1.1. Protective Equipment

A full body protective apiary suit, consisting of one-piece pants and jacket, hat, veil, and a pair of long-sleeve beekeeping gloves that was worn by the operators at the apiary when collecting *Vespa velutina* specimens. For venom removal, at the lab, it is necessary to use protective equipment: glasses, gloves, and mask to avoid contamination and potential direct contact with the skin and mucosas.

#### 3.1.2. The Vespa Velutina Venom Extraction Chamber

**Figure 10:**
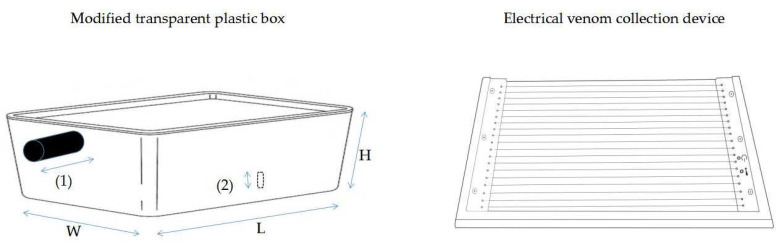
System set-up for *Vespa velutina* venom obtention chamber by electric stimulation.

System set-up for *Vespa velutina* venom obtention by electric stimulation consists of a modified container where a venom collection device is located (Figure 10), composed of:

(a) A transparent plastic box (285 × 160 × 120 mm) with a lid and hermetically sealed, in which modifications were made for the introduction of the captured insects (1) and to access the on/off switch of the electrical venom collection device with a stick (2). Two holes consisted of a circular, 30-mm diameter at a height of 70 mm from the base, for the introduction tube (1); and a rectangular aperture (10 mm × 20 mm) to give access to the on/off switch (2). A 100-mm-length plastic tubing, covered with grey adhesive tape, is inserted into hole number 1 of the plastic box with 45 mm protruding from the box and a screw cap at the end to prevent escape. Hole number two for the on/off switch mechanism is located at the back of the venom collection device, approx. 75–80 mm from the base of the box.

(b) An electrical venom collection device was supplied by IGK Electronics (Varna, Bulgaria), designed to harvest honey bee venom. The device consists of a solid wooden frame (250 × 158 × 38 mm) having an area 250 by 158 mm over which 39 wires are stretched at 3 mm intervals (Figure 10). A removable glass plate (201 × 140 × 4 mm) fits under the wires.

### 3.2. Collecting Insects and Venom

#### 3.2.1. Identification of Vespa Velutina Individuals

Insects were identified using their external morphological characteristics. In brief, *Vespa velutina* averages about 2–3 cm in length, the head is black with the face and mouthparts orange, and the antennae are brown dorsally and orange ventrally. The thorax is dark brown, almost black. Metasomal terga is brown, with a thin yellow band on the first segment and a thin orange band on the second and third segments; the fourth metasomal segment is orange; metasomal segments five and six are orange–brown. The legs are brown, with yellow tarsi, and the wings are brownish hyaline.

#### 3.2.2. Catching the Insects 

The collection of *Vespa velutina* specimens (n = 30) hovering in front of the beehives or the traps was performed by horizontally swinging the net quickly across the hornet, to capture the specimen and then follow through swipe to force the insect into the very bottom of the net bag tip. The above is performed with a fast twist of the wrist so that the bottom of the net bag hangs over the rim. If necessary, with the rim of the net in contact with the ground, hold the tip of the bag up with one hand. The *Vespa velutina* will fly or walk upward into the net at the tip of the bag, which can then be flipped over with the hand to entrap the specimen, keeping a sufficient amount of netting between the hand and insect.

#### 3.2.3. Transfer the Insects to the Venom Collection Chamber 

The trapped insect in a fold of the tip of the net is then inserted into the *Vespa velutina* venom extraction chamber. The part of the open net is placed embedded at the entrance of tube number 1, until we reach the pocket that we are holding with one hand. At this time, the inlet of the tube number one is surrounded with the net, so the insect has free access to the tube entry. Captured insects will crawl voluntarily toward the illuminated side in the venom collection box. Once the insect passes through the tube and enters the venom extraction box, which occurs in 3–5 s, the net is removed and the inlet tube is closed with the lid.

#### 3.2.4. Electric Stimulation 

The glass panels should be sterilized previously with 70–90% ethyl alcohol. Turn on the venom collector device with the switch On/Off key with a stick through hole number two. The device starts to work and you will see the green LED, flashing slowly: three times, pause, three times. Every minute the electrical device will pause for about 10 s. After about 40 min, the electrical venom collector turns itself off. Once the venom harvesting is complete, the insects (n = 10) are removed. Then, the *Vespa velutina* venom extraction chamber with the fresh *Vespa velutina* venom on the electrical venom collection device is carefully packed into a container for transportation to the laboratory. Three venom extraction sessions are performed.

#### 3.2.5. Vespa Velutina Venom Removal and Processing in the Laboratory

At the lab, the glass plate is pulled out from the electrical venom collection device and inspected visually under UV light. The glass plate is then scraped, with the help of a razor and spatula, and transferred to a vial. Venom scrapings were pooled. The dehydrated venom is then kept in dark bottles and stored in a refrigerator at −15 °C pending further analysis (see the Video S1).

### 3.3. ^1^H-NMR Analysis 

^1^H-NMR analyses were performed on a Varian VNMRS-500-WB spectrometer (500 MHz for ^1^H) instrument (Agilent Technologies, Palo Alto, CA, USA), equipped with a 5 mm probe. The sample was dissolved in 500 µL of methanol-d_4_ CD_3_OD, (Sigma-Aldrich, Madrid, Spain) shaken in a vortex mixer, and the resulting mixture was placed into 5-mm diameter ultra-precision NMR sample tubes (Norell, Landisville, PA, USA). The temperature of the sample in the probe was 30 °C. The chemical shifts are reported in ppm, using the solvent proton signal as standard. The area of the signals was determined by using the Mestrelab MNova (Mestrelab S.L. Spain, 2016) software, and the integrations were carried out three times to obtain average values. All figures of the ^1^H-NMR spectra and the expanded ^1^H-NMR spectrum regions were plotted at a fixed value of absolute intensity to be valid for comparative purposes.

Values for the peaks detected are as follows: ^1^H-NMR (500 MHz, CD_3_OD) δ 8.45, 8.38, 8.09, 8.05, 8.00, 7.97, 7.93, 7.87, 7.76, 7.75, 7.62, 7.61, 7.55, 7.51, 7.50, 7.29, 7.28, 7.23, 7.14, 7.13, 7.12, 7.12, 7.02, 7.01, 6.99, 6.98, 6.92, 6.90, 6.87, 6.86, 6.86, 6.67, 6.66, 6.65, 6.65, 3.90, 3.83, 3.74, 3.73, 3.72, 3.68, 3.62, 3.62, 3.61, 3.60, 3.40, 3.40, 3.38, 3.16, 3.15, 3.14, 3.13, 3.13, 3.10, 3.09, 3.08, 3.07, 2.99, 2.98, 2.97, 2.80, 2.52, 2.50, 2.49, 2.47, 2.18, 2.16, 1.97, 1.97, 1.93, 1.92, 1.90, 1.88, 1.86, 1.72, 1.70, 1.67, 1.62, 1.52, 1.48, 1.47, 1.45, 1.43, 1.42, 1.36, 1.34, 1.33, 1.32, 1.30, 1.02, 0.89, 0.89, 0.88, 0.85, 0.81, 0.80, 0.80, 0.79, 0.77, 0.77, 0.75, 0.73, 0.71, 0.62, 0.44, and 0.43.

## 4. Conclusions

We described a straightforward, quick, and inexpensive method for obtaining *Vespa velutina* venom, based on an electric stimulation protocol, including all sequential steps for sampling, handling, and milking the insect. The materials used are cheap and readily accessible in the market. The *Vespa velutina* venom extraction chamber can be used in field, since it is totally portable by operators with minimal training. Actually, the *Vespa velutina* venom extracts obtained by electro stimulation have allowed the initiation of new investigations in the Clinical Immunology Unit of the University Hospital of Santiago de Compostela, which we expect may ultimately promote substantial improvements in the diagnosis, prevention, and medical treatment of severe allergies and anaphylaxis caused by *Vespa velutina* stings. 

## Supplementary Materials

The following supporting information can be downloaded. Video S1: Scraped of the *Vespa velutina* Lepeletier 1836 venom obtained from a captive nest.

## Sample Availability

Samples of the *Vespa velutina* venom are available from the authors.
